# Physical Education Classes as a Precursor to the Mediterranean Diet and the Practice of Physical Activity

**DOI:** 10.3390/nu12010239

**Published:** 2020-01-16

**Authors:** Rubén Trigueros, Luis A. Mínguez, Jerónimo J. González-Bernal, José M. Aguilar-Parra, Raúl Soto-Cámara, Joaquín F. Álvarez, Patricia Rocamora

**Affiliations:** 1 Department of Psychology, Hum-878 Research Team, Health Research Centre, University of Almeria, 04120 Almeria, Spain; jalvarez@ual.es; 2 Department of Psychology, University of Burgos, 09001 Burgos, Spain; laminguez@ubu.es (L.A.M.); jejavier@ubu.es (J.J.G.-B.); rscamara@ubu.es (R.S.-C.); 3 Department of Nursing, Physiotherapy and Medicine, Health Research Centre, University of Almería, 04120 Almería, Spain; rocamora@ual.es

**Keywords:** adolescence, mediterranean diet, physical activity, physical education, resilience, motivation

## Abstract

Physical activity and a healthy, balanced diet are remaining unresolved issues among young people. According to the World Health Organization, young people do not get enough exercise during the week, and physical education classes are the best way to promote healthy habits. This study aims to analyze how the role of the teacher influences the frustration of psychological needs, coping strategies, motivation, and the adoption of healthy eating habits through the Mediterranean diet and the regular practice of physical activity. The study involved 1031 boys and 910 girls between the ages of 13 and 18. To explain the relationships between the different variables included in this study, a model of structural equations has been developed. The results showed that autonomy support negatively predicted the frustration of four psychological needs. The failure to meet four psychological needs negatively predicted resilience. Likewise, resilience positively predicted autonomous motivation, and this positively predicted the Mediterranean diet and the practice of physical activity. Thus, the results obtained in the present study are in line with those of various studies wherein physical education classes were seen to help consolidate healthy living habits.

## 1. Introduction

Adolescence is a transitional stage in which there are important biological, psychological, and social changes that can affect young people’s well-being [1]. It is during this period of life that young people begin to have greater independence and make their first independent decisions, establishing many of the behaviors that they will maintain during adulthood [2,3]. Thus, it is during adolescence that physical activity begins to decrease as they prefer to do other types of activities, and young people begin to eat unhealthy foods [4]. Therefore, in recent years, with the emergence of the science of positive psychology [5], new models have emerged that, starting from a deficit-based approach, move toward a new paradigm focused on the optimal functioning of adolescents [6]. These models of positive development attempt to determine the factors that promote healthy development during adolescence, with the adoption of healthy lifestyle habits and the regular practice of physical activity being two of the main aspects on which they focus [7].

### 1.1. Self-Determination Theory

Self-determination theory (SDT) is a social theory that tries to explain the influence of the social environment on people’s adaptive and nonadoptive behaviors [8]. In this sense, the SDT affirms that the influence of the social context turns out to be key for the development of certain behaviors in individuals and that this can occur through two antagonistic interpersonal styles, controlling style versus autonomy support [9]. The first assumes that the social environment tries to influence the behavior of the person through the use of punishment, threats, obligations, abuses, and so forth, being observed by the individual as the cause of their behaviors [10]. This implies the loss of self-initiative and personal effort. On the other hand, autonomy support refers to the physical and mental self-development of the individual and to one’s own initiative, with the support of the social environment and without eternal pressures [9]. Depending on the teacher, the choice of interpersonal style will influence the psychological needs of students in a significant way [8].

Starting from the postulates of SDT, psychological needs can be understood as essential elements that promote personal well-being [11]. These psychological needs include competence, which refers to feeling capable when performing any activity; autonomy, which is defined as the desire to feel that behavior originates with oneself; and relatedness, which refers to feeling integrated and understood by the social reference group [12]. In recent years, starting with the studies of González-Cutre et al. [13] and Trigueros et al. [14], it has been proposed that we incorporate a fourth psychological need called novelty, which is linked to the individual’s search for new activities and experiences that will contribute to personal well-being.

In this way, students who feel autonomous and competent when they make the decision to participate in a given activity, and who feel integrated and supported by the social reference group and view the activities as interesting and different, will experience the satisfaction of their psychological needs, which, in turn, will significantly influence the autonomous motivation of the individual, which is related to learning new skills, psychological well-being, commitment, effort, and the adoption of adaptive behaviors [8,9]. On the other hand, if students feel controlled in their behaviors, experience a feeling of abandonment, or view the class challenges as complicated or repetitive, they will feel that their psychological needs are thwarted due to experiencing controlled motivation or even amotivation [10]. This type of motivation is generally linked to a lack of commitment to activity, abandonment, poor interpersonal relationships, and the adoption of nonadaptive behaviors [15].

In this way, psychological needs, as well as the social context, can have a significant influence on the student’s motivation towards PE classes [16,17]. Following SDT, motivation can be controlled motivation or autonomous. The first is related to the acquisition of external obligations or rewards when performing an activity. On the contrary, autonomous motivation is linked to one’s own choice and internal commitment when carrying out any activity. The latter leads to the self-regulation of the individual’s behavior, remaining in the activity due to the internal satisfaction it produces. However, controlled motivation facilitates nonadaptive behaviors in that the student tends to move away from the activity if the obligation disappears and/or is not rewarded [18].

Current studies in the field of physical education classes are primarily interested in the clear perspective of SDT, examining the influence of autonomy support, the satisfaction of psychological needs, and autonomous motivation on the adoption of behaviors conducive to a healthy lifestyle [19]. Therefore, in recent years, a new line of research has emerged that has focused on the dark perspective of SDT, since PE classes can be an environment that can generate behaviors contrary to the adoption of healthy lifestyle habits if the controlling style of the teacher leads to the frustration of psychological needs [20], generating negative effects on autonomous motivation [21], learning [22], metacognitive strategies [23], and emotional intelligence [24]. Few studies have even looked at the dark side of SDT, or taken into consideration autonomy support versus the controlling style.

### 1.2. Resilience

Among the psychological factors affecting personal well-being is resilience, which refers to a set of positive psychological qualities linked to individual adaptation to adverse circumstances through the use of positive coping strategies [25]. In this sense, PE classes, unlike other academic areas, are characterized by continuous exposure to a series of adverse and stressful experiences that students must confront at some point (difficult situations, injuries, friction with classmates and/or teachers, etc.) [24]. In this way, the area of PE contributes to the development of human capacity to face, overcome, and come out stronger from those experiences of adversity.

These adverse situations and their overcoming were initially studied through the metatheory of resilience [26]. This theory affirms that resilience begins in a situation of physical, mental, and spiritual balance that is interrupted when a given situation arises where the individual does not possess sufficient resources or abilities to face adverse situations [27]. Over time, the individual will readjust and regain balance by raising his level of resilience or homeostasis. However, this metatheory suffers from a series of limitations since it considers resilience as a linear model, whereby the individual faces a single event. Furthermore, the model does not explain how emotions can affect the process of overcoming. This is especially important given the protective nature of emotions in the behavior of individuals [28] and the fact that those who show a capacity for resilience value emotions as facilitators to achieve it [29].

Because of these limitations, Fletcher and Sarkar [30] tried to reconceptualize the definition of resilience as being linked to the possession and presence of protective and vulnerability factors within and outside the people, which encourage the helpful adaptation to risk. Thus, the protective factors of resilience have been explored through numerous research trying to identify the qualities of resilient individuals in the fields of health [31], work [32], and sports [33], and there are a few studies in the educational field (e.g., [34]), focused mainly on external aspects of students rather than on what happens during classes and the influence it exerts with respect to the psychological responses it generates in students.

These resilience models propose a comprehensive representation of the process and outcome of resilience during the process of adaptation to difficulties. Furthermore, these models allow their application in psychoeducational interventions for the prevention of risk behaviors.

### 1.3. Objective and Hypothesis

As stated above, the aim of this study is to analyze how the role of the teacher influences the frustration of psychological needs, coping strategies, motivation, and the adoption of healthy eating habits through the Mediterranean diet and regular practice of physical activity. The following hypotheses have been formulated: (**1**) autonomy support will negatively predict the frustration of psychological needs, while psychological control will positively predict it; (**2**) frustration of psychological needs will negatively predict resilience; (**3**) resilience will positively predict autonomous motivation; (**4**) autonomous motivation will positively predict the adoption of a healthful Mediterranean diet and the practice of physical activity.

## 2. Materials and Methods

### 2.1. Participants

The secondary school students participating in the study were 1031 boys and 910 girls, for a total of 1941 students. The age of the students ranged from 13 to 18 years (M = 15.34; SD = 1.22). The PE classes were held with equal rights and duties between students belonging to various secondary educational centers in Almeria and Burgos.

Informed consent from parents or legal guardians as well as voluntary participation in the study were the criteria for the inclusion of students in the study.

### 2.2. Instruments

Perceived autonomy support. The Scale used was the Perceived Autonomy Support Scale for Exercise Settings (PASSES; [35]), validated for the Spanish PE context [36]. The scale evaluates a single factor called autonomy support through 12 items. The scale ranges from totally disagreed (1) to totally agreed (7).

Psychological controlling. The scale used was a version of the Psychologically Controlling Teaching Scale (PCTs; [37]), validated and adapted for the PE context [38]. The instrument consists of seven items with a single factor. The scale ranges from totally disagreed (1) to totally agreed (7).

Frustration of psychological needs: The scale utilized was the scale of Frustration of Psychological Needs, validated and adapted for the Spanish PE context [39]. The instrument consists of 17 items, distributed as follows among each of the factors that make up the scale: four items for competence, four items for relatedness, four items for autonomy, and five items for novelty. The scale ranges from not at all true (1) to totally true (7).

Resilience in PE. The instrument utilized was the Resilience Scale in PE classes [40]. This questionnaire consists of 25 items divided between two factors that measure acceptance of oneself and the context and personal competence. The scale ranges from total disagreement (1) to total agreement (7).

Motivation. The instrument utilized was the Perceived Locus of Causality Revised (PLOC-R; [41]), validated and adapted for the Spanish context of PE [42]. The questionnaire consists of 23 items divided into six factors that measure different kinds of motivation. The scale ranges from not at all true (1) to totally true (7).

For this study, the self-determination index (SDI) was calculated to quantify the level of self-determination [43] using the following formula: 3 × intrinsic motivation, 2 × integrated regulation, 1 × identified regulation, −1 × introjected regulation, −2 × external regulation, and −3 × demotivation.

Practice of physical activity. The Spanish version [44] of the WHO Health Behavior of Schoolchildren Survey [45] was used. For this study, we selected indices that referred to the practice of physical activity; an index was calculated based on the number of days per week of each physical activity and the duration of the sessions. For a more detailed explanation of the indices and their validity, see Balaguer [44].

Mediterranean diet. The kidmed scale [46], which measures dietary patterns related to the Mediterranean diet, was used. This scale has an index oscillating from 0 to 12 across 16 questions. Those questions with a negative connotation with respect to the Mediterranean diet were assigned a value of −1, and those with a positive connotation were assigned +1.

### 2.3. Procedure

Once the questionnaires were selected, several educational centers were contacted whose PE teachers were briefed of the aims of the present study in relation to resilience, motivation, and the adoption of healthy dietary patterns and the practice of physical activity. Subsequently, those students who wished to participate in the present study were required to obtain written authorization from their parents or legal guardians, since they were minors. The scales were given before the beginning of PE classes during the third week of February of the academic year 2018/2019, with the answers to the questionnaires being anonymous.

El estudio se llevó a cabo en cumplimiento de las directrices de la Asociación Psicológica Americana. The Research Ethics Committee of the University of Almeria, Spain, approved the present study (Ref. UALBIO 2019/014).

### 2.4. Data Analysis

In this study, the statistical program SPSS 25 was used to perform different analyses (e.g., mean, standard deviation, bivariate correlations, and reliability). In addition, the statistical program AMOS 20 was used to create a structural equation model (SEM).

**Figure 1:**
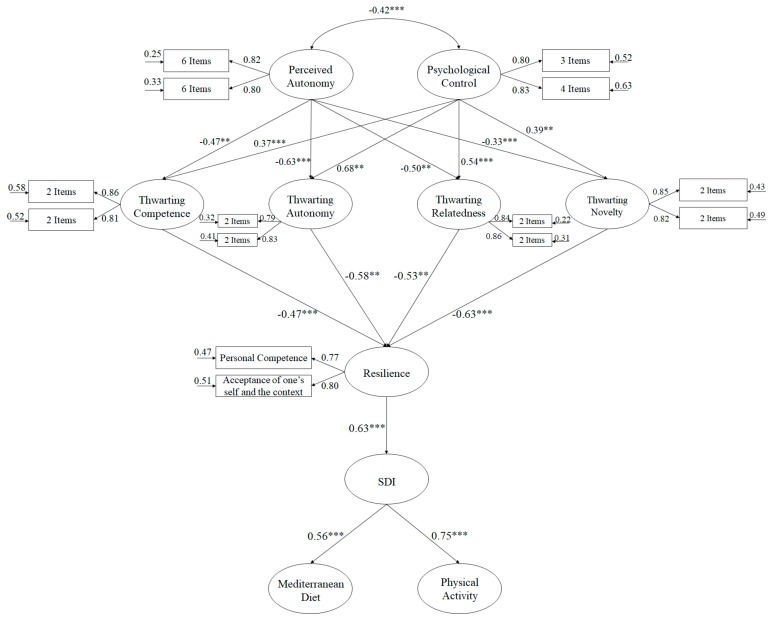
Hypothesized model, analizado a través de un SEM. All parameters are statistically significant. Note: *** *p* < 0.001; ** *p* < 0.01. SDI: self-determination index.

With the purpose of analyzing the hypothetical model (Figure 1), the bootstrapping procedure was used, together with the maximum likelihood method. The estimators were considered robust despite the lack of normality. To judge the model tested, several adjustment rates were examined: values of *χ*^2^/df lower than 3, comparative fit index (CFI) and incremental fit index (IFI) values close to or higher than 0.95, and root mean square error of approximation (RMSEA) and standardized root mean square residual (SRMR) values lower than or very close to 0.06 and 0.08, respectively [47], were considered indicative of the adequate fit of the model. However, for complex models these adjustment fit rates should be interpreted with care as it is very restrictive [48].

## 3. Results

### 3.1. Preliminary Analyses

Table 1 presents analyses of mean and standard deviation, bivariate correlations through Pearson, and reliability analysis through Cronbach’s α of all variables supporting autonomy, psychological controlling, frustration of competition, relatedness, autonomy, novelty, resilience, SDI, adoption of the Mediterranean diet, and practice of physical activity.

As for the correlation analyses, these reflected a positive correlation between autonomy support, SDI, resilience, adoption of the Mediterranean diet, and physical activity practice, as well as negative correlations regarding psychological controlling, frustration of competition, relatedness, autonomy, novelty, and adoption of the Mediterranean diet.

### 3.2. Structural Equations Model

Due to the complexity of the hypothesized model (Figure 1), the number of indicators was reduced to at least two, in order to be able to analyze the relationships between the study variables [49]. In particular, the latent variables considered were resilience, which included two indicators (personal competence and acceptance of self and context) [39]; autonomy support, divided into the 12 items of the scale using two indicators, including the seven items of psychological control, the four items of frustration of competence, relatedness, autonomy, and novelty, all in order to identify the model [49].

The hypothesized predictive relationships model (Figure 1) showed that the adjustment indices were satisfactory: *χ*^2^ (106, N = 1941) = 241.52, *χ*^2^*/df* = 2.28, *p* < 0.001, IFI = 0.95, CFI = 0.95, RMSEA = 0.053 (Confidence interval 90% = 0.049–0.058), SRMR = 0.048. These results were adjusted to the established parameters, so the proposed model was accepted as adequate. In addition, the contribution of each of the factors to the prediction of other variables was examined through standardized regression weights.

## 4. Discussions

This study has attempted to analyze the way in which the interpersonal style of the teacher (autonomy support versus psychological controlling) influences the thwarting of psychological needs, resilience, autonomous motivation, and behaviors related to the adoption of balanced eating habits, which are typical of the Mediterranean diet, and the practice of physical activity.

This study examines for the first time the dual role of the teacher in each of the factors belonging to the thwarting of psychological needs, with special emphasis on the novelty factor as it is a psychological need of recent incorporation. In this sense, the studies that have analyzed the influence of the teacher based on the psychological needs of students have focused only on the bright side, that is, on the influence of autonomy support on the satisfaction of psychological needs, and the dark side is still under-represented in the literature [50,51]. On the other hand, the present study contemplates for the first time the influence of the dark side of SDT, that is, psychological control and the frustration of needs, on resilience in the field of Physical Education. In this sense, analyzing the dual role of the teacher is relevant given the influence it has on the social, emotional, and psychological development of students [52]. In addition, it is important to highlight the influence that teachers have on students’ development of skills and knowledge based on a balance between discovery and previous experiences, which allows them to overcome difficulties and make the best decisions related to the adoption of behaviors that promote health [53,54]. In addition, PE is a discipline where students are exposed to continuous stressful situations and where, through the different proposed activities, they feel continually challenged, so success and failure are often present during classes [55,56,57]. Such circumstances can influence the opinion they have of PE classes, assuming a greater or lesser involvement of students during PE classes, affecting in the final stage the development of behaviors aimed at the adoption of healthy lifestyle habits.

The results of the present study showed how psychological control positively predicted the thwarting of the psychological needs of autonomy, competence, relatedness, and novelty; on the contrary, autonomy support negatively predicted each of the thwarting frustrations. These results cannot be compared with similar studies because we have no evidence of research that has analyzed this relationship in the context of physical education, especially in relation to the thwarting of novelty. However, there is evidence from studies that have analyzed the influence of autonomy support on the satisfaction of psychological needs, and in recent years, on novelty. A study with high school students conducted by Zhang, Solmon, Kosma, Carson, and Gu [58] showed that those students who had high levels of support for their autonomy were positively related to each of the factors of satisfaction of psychological needs, except novelty. However, a recent study by González-Cutre, Romero-Elías, Jiménez-Loaisa, Beltrán-Carrillo, and Hagger [59] showed how support for autonomy was positively related to each of the factors of psychological needs, including novelty. Similarly, different studies in the field of PE (i.e., [60,61,62,63]) have shown how support for autonomy predicted the thwarting of psychological needs in a negative way; on the contrary, psychological control predicted them in a positive way, but the thwarting of psychological needs was analyzed in a global way and not through the factors that compose it. Thus, the results of the present study are in line with the results shown in previous studies and within the postulates of SDT. The results on the dual role of the teacher and the frustrations of the psychological needs established in this study can be explained by the fact that if students feel coerced, rejected, and limited in their decision-making, they will feel frustrated in their perceived autonomy and competence, in their psychological well-being, and in their psychological needs.

The results also show that frustration of the four psychological needs of competence, autonomy, relatedness, and novelty negatively predicts resilience. These results are similar to those of various studies in the field of sports, where the negative effect of the frustration of psychological needs in relation to resilience has been observed, in contrast to the positive influence of satisfaction [64,65]. However, there is little evidence of research that has analyzed the influence of the four factors of frustration of psychological needs on student resilience in the context of PE. In this sense, the present study shows the importance of creating a climate where students feel their psychological needs are satisfied, in order to promote the adaptability of the students to the multiple vicissitudes that present themselves while participating in the different PE classes. To this end, it is essential that teachers try to instill in their students personal skills, social competence, autonomy, optimism, and hope [66].

Finally, the results revealed that resilience positively predicted autonomous motivation. However, studies on resilience in the field of PE classes are scarce, and there is little evidence of studies that have analyzed this relationship. Despite this situation, there are some studies in the university setting [67] that have analyzed the influence of resilience on autonomous motivation that indicated that those students who are psychologically resistant (resilient) use internal coping strategies that lead them to perceive, access, and precisely regulate their behaviors in order to achieve their objectives (autonomous motivation). Furthermore, in the sporting context, there are several studies that have explored this relationship. A study carried out by Sarkar and Fletcher [27] with semiprofessional athletes showed that those who had high levels of resilience showed a higher predisposition towards exercise merely for improving their own capacities and abilities through the overcoming of challenges. In addition, the present study has shown how autonomous motivation towards PE classes acted as a predictor of the adoption of a Mediterranean diet and regular physical activity. Although no studies have previously analyzed the relationship between motivation and the Mediterranean diet, a study by Jiménez, Cervelló, García, Santos, and Iglesias [68] with adolescents showed that those who actively participated in Physical Education classes were more predisposed to physical activity and a healthy, balanced diet. In such a way, the results of this work suggest that if physical education classes facilitate the adaptability and motivation of the students, they will feel more predisposed towards the assimilation of contents, abilities, and attitudes that focus on the carrying out of physical activity and maintaining a healthy and balanced diet typical of the Mediterranean [69].

There are several limitations to this study that must be highlighted. First, it is based primarily on self-reported measures. Secondly, it is a relational study that does not allow for the extrapolation of cause‒effect relationships, so that the results obtained can be interpreted in different ways, based on the individual’s viewpoint. On the other hand, future studies should analyze the influence of the social context on the resilience levels of students as well as on well-eating habits and regular practice of physical activity, given that it is a time of multiple changes for adolescents.

## 5. Conclusions

The results of the present study are in line with the theoretical postulates referring to the dark side of the SDT, demonstrating the importance and influence of the context of PE classes in the adoption of a Mediterranean diet and the regular practice of physical activity. In order to do so, teachers must create educational programs that focus on the achievement of basic objectives in the area of PE, based on positive experiences focused on the resolution of complex motor skills.

## Figures and Tables

**Table 1:** Preliminary analyses and correlations.

Factors	M	SD	α	1	2	3	4	5	6	7	8	9	10
1. Autonomy Support	4.65	0.98	0.84	−	−0.47 ***	−0.52 **	−0.75 ***	−0.31 ***	−0.38 ***	0.21 **	0.42 ***	0.30 **	0.17 **
2. Psychological Controlling	1.68	1.24	0.81		−	0.28 **	0.32 **	0.49 **	0.52 **	0.61 **	0.25 **	−0.37 ***	−0.52 ***
3. T. of Competence	5.23	0.78	0.80			−	0.71 ***	0.58 **	0.77 **	−0.72 ***	−0.23 ***	−0.31 **	−0.31 **
4. T. of Autonomy	5.41	0.43	0.83				−	0.56 ***	0.61 ***	−0.61 **	−0.51 **	−0.37 **	−0.42 ***
5. T. of Relatedness	5.01	0.94	0.88					−	0.53 **	−0.59 ***	−0.35 **	−0.22 *	−0.37 **
6. T. of Novelty	4.95	0.86	0.87						−	−0.36 ***	−0.42 **	−0.25 **	−0.51 ***
7. Resilience	5.89	1.17	0.92							−	0.67 ***	0.59 **	−0.35 **
8. SDI	14.67	12.93	−								‒	0.33 ***	−0.59 ***
9. Mediterranean Diet	8.52	1.77	−									‒	−0.60 ***
10. Physical Activity	4.32	1.35	−										−

Note: T = Thwarting; SDI = Self-Determination Index; *** *p* < 0.001; ** *p* < 0.01; * *p* < 0.05.
